# Secreted protein acidic and rich in cysteine internalization and its age-related alterations in skeletal muscle progenitor cells

**DOI:** 10.1111/acel.12168

**Published:** 2013-11-19

**Authors:** Katsuyuki Nakamura, Keitaro Yamanouchi, Masugi Nishihara

**Affiliations:** Department of Veterinary Physiology, Graduate School of Agricultural and Life Sciences, The University of Tokyo1-1-1 Yayoi, Bunkyo-ku, Tokyo, 113-8657, Japan

**Keywords:** adipogenesis, differentiation, endocytosis, integrin-α5, secreted protein acidic and rich in cysteine, skeletal muscle

## Abstract

Aging causes phenotypic changes in skeletal muscle progenitor cells (Skm-PCs), such as reduced myogenesis and increased adipogenesis due to alterations in their environment or niche. Secreted protein acidic and rich in cysteine (SPARC), which is secreted into the niche of Skm-PCs, inhibits adipogenesis and promotes myogenesis. We have previously reported that Skm-PC responsiveness to SPARC declines with age, although the mechanism underlying this decline is unknown. In this study, we found that SPARC is internalized by Skm-PCs and that this uptake increases with age. Internalization is dependent on integrin-α5, a cell surface SPARC-binding molecule, and clathrin-mediated endocytosis. We also demonstrated that internalized SPARC is transported to Rab7-positive endosomes. Skm-PCs from old rats exhibited increased clathrin expression and decreased Rab7 expression exclusively in MyoD-negative cells. In loss-of-function analyses, clathrin knockdown increased the anti-adipogenic effect of SPARC, whereas Rab7 knockdown reduced it, indicating that alterations in SPARC internalization may mediate the age-related decline in its anti-adipogenic effect. These results provide insights into age-related SPARC resistance in Skm-PCs, which may lead to sarcopenia.

## Introduction

Age-related degeneration of skeletal muscle, known as sarcopenia, is characterized by loss of lean muscle mass and declining muscle function (Forbes & Reina, 1970; Thomas, 2010), leading to falls and dependency. Intramuscular adipose tissue often increases in elderly individuals (Visser *et al*., 2002; Song *et al*., 2004), causing harmful effects such as insulin resistance (Zoico *et al*., 2010).

Skeletal muscle comprises several progenitors. Myogenic progenitors differentiate from tissue-specific stem cells called satellite cells (Mauro, 1961), which exist in a quiescent state between the sarcolemma and the basal lamina. Recent studies have characterized satellite cell populations in skeletal muscle with satellite cell-specific markers such as SM/C-2.6 (Fukada *et al*., 2004) or integrin-α7 (Sacco *et al*., 2008). By using these satellite cell-specific markers of SM/C-2.6 and integrin-α7, researchers have separated the population of adipogenic progenitors in skeletal muscle from satellite cells and have identified the progenitors as PDGFRα-positive or Sca-1-positive cells, called fibrocyte/adipocyte progenitors (FAPs, Joe *et al*., 2010; Uezumi *et al*., 2010). FAPs themselves do not participate in myotube formation, but they can differentiate into adipocytes both *in vitro* and *in vivo*. In general, enzymatically isolated and primary cultured cells from skeletal muscle [skeletal muscle-derived progenitor cells (Skm-PCs)] contain both myogenic and adipogenic progenitors. The myogenic progenitors can be identified immunochemically for the expression of Pax7 and/or MyoD. The adipogenic progenitors can give rise to PPARγ-positive and/or Oil Red-O-stained adipocyte upon adipogenic stimulation. It has been reported that the degree of adipogenesis of Skm-PCs upon adipogenic stimulation increases with age (Taylor-Jones *et al*., 2002). This is in agreement with the finding that intramuscular adipose tissue content is higher in old mice (Liu *et al*., 2012) and in elderly individuals (Song *et al*., 2004).

A parabiosis study, in which the circulatory systems of young and old mice were bound, demonstrated that the microenvironment or niche mediate the age-related dysfunction in myogenic progenitors (Conboy *et al*., 2005). Age-related increase in fibrosis after injury is also ameliorated by exposure to the blood of young mice (Brack *et al*., 2007). [Correction added on 7 January2014, after first online publication: A sentence referencing to a retracted article (Mayack *et al*., 2010) has been removed.] Thus, age-related alteration of the niche is a critical contributor to aging in skeletal muscle, and these alterations likely lead to changes in the behavior of progenitor cells. The niche also regulates the adipogenic program of Skm-PCs; for example, degradation of the basal lamina of myofibers, a major component of the niche, promotes adipogenic differentiation of Skm-PCs (Hosoyama *et al*., 2009).

Secreted protein acidic and rich in cysteine (SPARC; also known as osteonectin) is a 43-kDa matricellular glycoprotein that is secreted into the niche and functions in cell adhesion, angiogenesis, growth factor binding, and cell differentiation in various organs and cell types (Brekken & Sage, 2001; Kos & Wilding, 2010). SPARC binds integrin-α5 (Nie *et al*., 2008) and inhibits adipogenesis of pre-adipocytes isolated from murine adipose tissue by enhancing β-catenin signaling through integrin-linked kinase (Nie & Sage, 2009). SPARC also promotes myogenic differentiation of C2C12 and MM14 myoblasts (Cho *et al*., 2000; Motamed *et al*., 2003). We previously reported that although SPARC expression in Skm-PCs does not change, Skm-PCs become refractory to SPARC with age (Nakamura *et al*., 2012). We suggested that the anti-adipogenic effect of SPARC occurs through integrin-α5, whose expression is reduced in Skm-PCs from old rats.

Integrins on the cell membrane mediate cellular–ECM interactions; their activity is regulated by various intracellular and intercellular signals, some of which are internalized through endocytic pathways. Once internalized, integrins are recycled into the plasma membrane by recycling endosomes (Caswell *et al*., 2009). In general, a ligand is internalized through receptor-mediated endocytosis or pinocytosis, which is categorized into macropinocytosis and clathrin-mediated or caveolin-mediated pathways (Conner & Schmid, 2003). Integrins are internalized through macropinocytosis (Gu *et al*., 2011), as well as through clathrin-mediated (Ezratty *et al*., 2009) and caveolin-mediated pathways (Caswell & Norman, 2006). Integrin internalization is an important regulator of cell differentiation in mesenchymal stem cells from the rat bone marrow (Du *et al*., 2011). Thus, we hypothesized that internalization is also involved in the differentiational regulation mediated by SPARC interaction with integrin-α5.

In this study, we tested the hypothesis that SPARC is internalized and characterized the age-related changes that occur in this process.

## Results

### SPARC internalization in rat Skm-PCs increases with age

**Figure 1:**
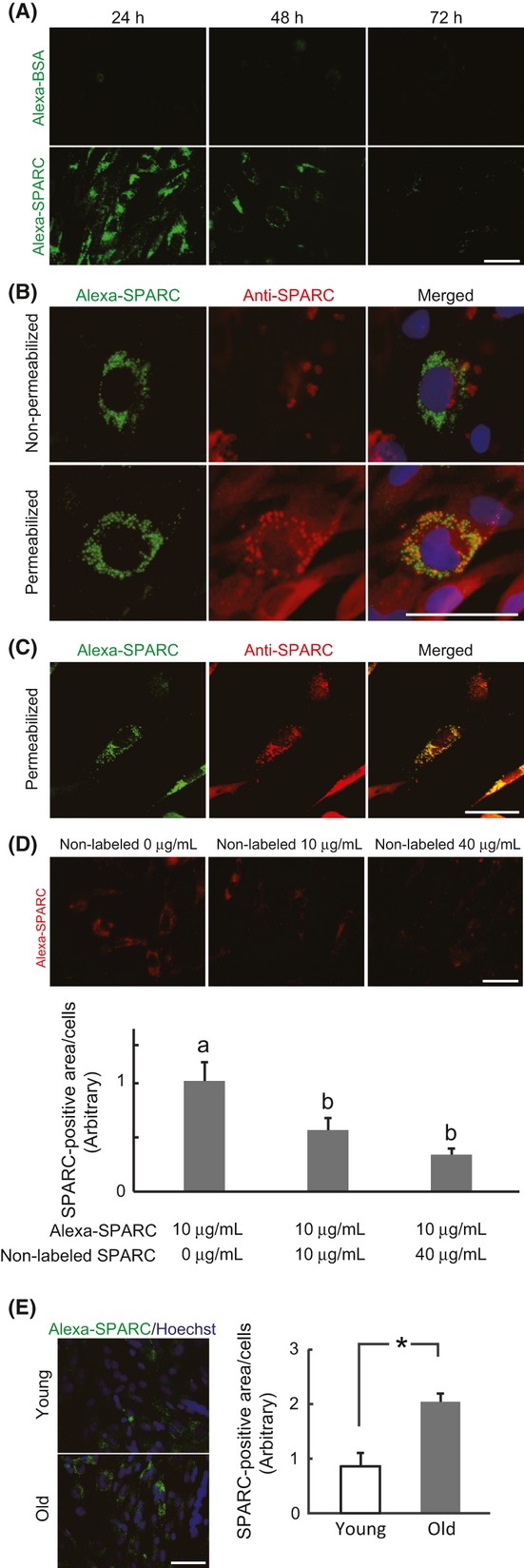
Internalization of SPARC in rat Skm-PCs and age-related changes. (A) Skm-PCs from young rats were cultured with 20 μg/mL Alexa-conjugated BSA (Alexa-BSA, above) or SPARC (Alexa-SPARC, below) for 24 h and incubated for 1 or 2 days. Times from the addition of Alexa-SPARC to Skm-PC fixation are indicated above the photographs. Scale bar = 50 μm. (B) Immunostaining of SPARC (red) was performed after incubation with Alexa-SPARC (green) with (below) or without (above) Trion-X permeabilization. Nuclei were visualized with Hoechst 33258 dye (blue). Scale bar = 50 μm (C) Skm-PCs from young rats were incubated with Alexa-SPARC (green) and immunostained with anti-SPARC antibody (red) after permeabilization; photographs were obtained by confocal microscopy. Scale bar = 40 μm. (D) Alexa-SPARC (red) was competed with non-Alexa-labeled SPARC in Skm-PCs from young rats for 12 h. Ten cells were randomly chosen, and SPARC fluorescence per cell was quantified by ImageJ. Scale bar = 50 μm. Error bars represent means ± SEM (*n* = 10 cells, respectively). Bars sharing the same letters are not significantly different. *P* < 0.05 (Tukey-Kramer’s test). (E) The photographs represent Skm-PCs from young and old rats incubated with Alexa-SPARC (green) for 12 h. Nuclei were visualized with Hoechst 33258 dye (blue). Scale bar = 50 μm. The amount of SPARC internalized into Skm-PCs was quantified by ImageJ and compared between young and old rats. Error bars represent means ± SEM (*n* = 3, respectively). *P < 0.05. Skm-PCs, skeletal muscle progenitor cells; SPARC, secreted protein acidic and rich in cysteine.

To visualize the distribution of SPARC in Skm-PCs, recombinant Alexa-Fluor-conjugated SPARC was added to cultures of young Skm-PCs for 24 h (Fig. 1A). The Alexa-Fluor-conjugated BSA control was minimal, whereas Alexa-SPARC was observed in the perinuclear area, suggesting its internalization. Fluorescence decreased over time after removal of the exogenous Alexa-SPARC. To further confirm the internalization of Alexa-SPARC, we performed immunostaining of SPARC under both permeabilized (treated with Triton-X, Sigma, St. Louis, MO, USA) and nonpermeabilized (without Triton-X) conditions. Under permeabilized condition, Alexa-SPARC within the cytoplasm was immunolabeled by anti-SPARC antibody, whereas under nonpermeabilized conditions, it was not labeled, indicating that Alexa-SPARC is indeed localized within the cell (Fig. 1B). Confocal microscopy confirmed the staining of Alexa-SPARC by anti-SPARC antibody under permeabilized conditions (Fig. 1C). To examine SPARC internalization through the SPARC-specific pathway, Alexa-SPARC and nonlabeled SPARC were added to the media for 12 h (Fig. 1D). The levels of internalized Alexa-SPARC decreased in the presence of 10 and 40 μg/mL nonlabeled SPARC, indicating the presence of a specific internalization pathway. When SPARC internalization was compared between Skm-PCs from young and old rats 12 h after the addition of Alexa-SPARC, internalized SPARC levels were greater in Skm-PCs from the old rats (Fig. 1E). These results suggest that SPARC is internalized through a SPARC-specific pathway and that its internalization increases with age.

### Integrin-α5-dependent SPARC internalization

**Figure 2:**
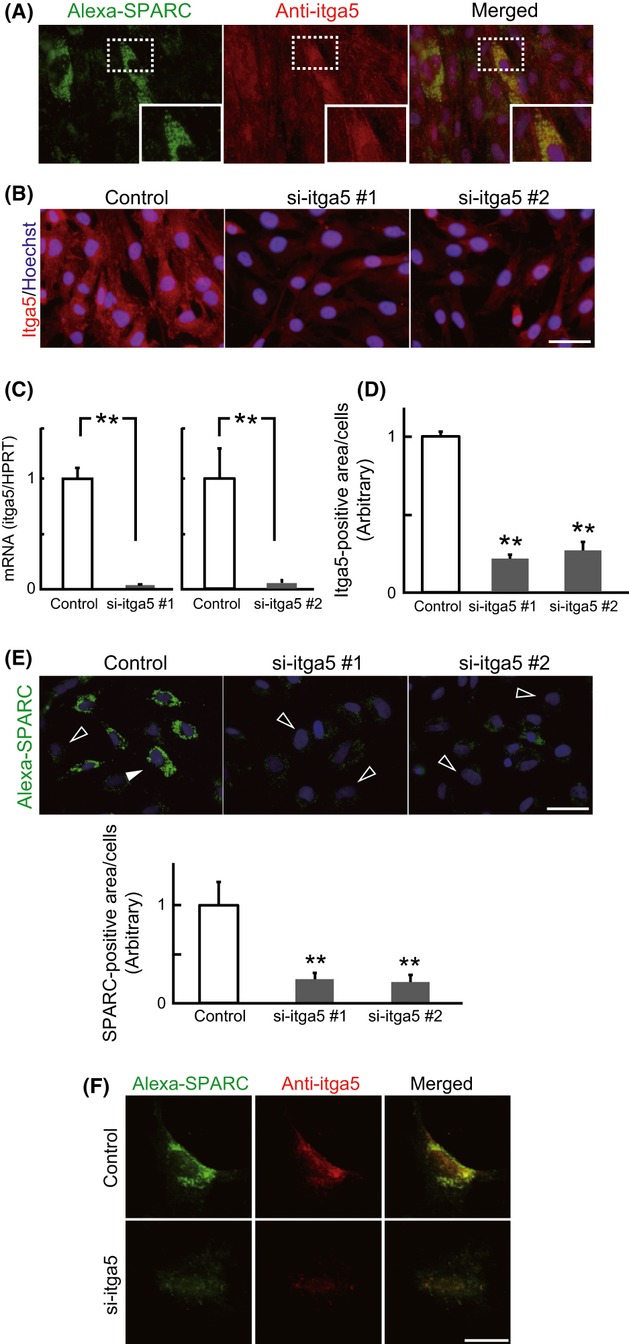
Integrin-α5-dependent internalization of SPARC. (A) Skm-PCs from young rats were incubated with Alexa-SPARC (green) and immunostained with anti-itga5 antibody (red). Insets of structures in the dash-lined boxes are shown enlarged in the bottom right corners. Nuclei were visualized with Hoechst 33258 dye (blue). (B) Representative images of itga5-targeted siRNA (si-itga5) in Skm-PCs 2 days after transfection. Scale bar = 50 μm. (C) si-itga5 silencing efficiency was evaluated by qPCR in Skm-PCs from young rats 2 days after siRNA transfection. Data are expressed as the means ± SEM (*n* = 3). **, *P* < 0.01 vs. control. (D) si-itga5 silencing efficiency was evaluated by immunostaining in Skm-PCs from young rats 2 days after siRNA transfection. Data are expressed as the means ± SEM (*n* = 3). **, *P* < 0.01 (Dunnett’s test). (E) SPARC fluorescence per cell was quantified by ImageJ and compared between control and si-itga5 groups. Black arrowheads indicate weakly and white arrowheads indicate strongly SPARC-internalizing cells, respectively. Scale bar = 50 μm. Error bars represent means ± SEM (*n* = 10 cells). **, *P* < 0.01 (Dunnett’s test). (F) Confocal images of Skm-PCs from young rats incubated with Alexa-SPARC and immunostained with anti-itga5 antibody 2 days after si-itga5 transfection. Scale bar = 20 μm. Skm-PCs, skeletal muscle progenitor cells; SPARC, secreted protein acidic and rich in cysteine.

SPARC binds integrin-α5 (Nie *et al*., 2008), and integrin internalization regulates mesenchymal stem cell differentiation (Du *et al*., 2011). Cell-associated Alexa-SPARC co-localized with integrin-α5 (itga5, Fig. 2A). To verify itga5-mediated SPARC internalization, itga5 expression was suppressed by siRNA before the addition of Alexa-SPARC. Itga5-targeted siRNA suppressed approximately 95% of itga5 gene expression (Fig. 2C) and 70% of itga5 protein expression (Fig. 2B and D). Under these conditions, quantitative analysis revealed that 80% of internalized SPARC was eliminated by si-itga5 (Fig. 2E). Confocal microscopy also showed the similar result that suppression of itga5 reduced Alexa-SPARC; in the control, SPARC co-localized with itga5 (Fig. 2F). Therefore, we suggest that SPARC interacts with itga5 and is internalized in Skm-PCs.

### Clathrin-dependent SPARC internalization

**Figure 3:**
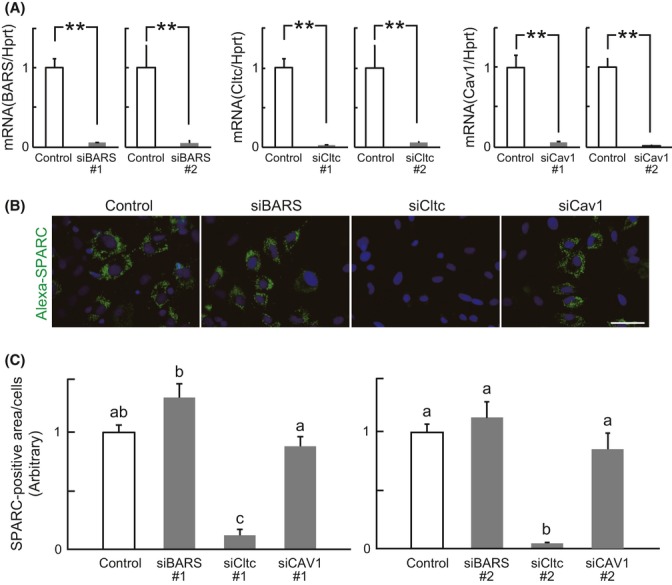
Clathrin-mediated SPARC internalization. (A) The efficiency of siRNA targeted to BARS (siBARS), clathrin heavy chain (siCltc), and caveolin1 (siCav1) was evaluated by qPCR in Skm-PCs from young rats 2 days after siRNA transfection. Error bars represent means ± SEM (*n* = 3, respectively). **, *P* < 0.01. (B) Representative images of Skm-PCs from young rats incubated with Alexa-SPARC after siBARS, siCltc, and siCav1 transfection. Scale bar = 50 μm. (C) SPARC fluorescence per cell was quantified by ImageJ and compared between groups. Error bars represent means ± SEM (*n* = 10 cells). Bars sharing the same letters are not significantly different. *P* < 0.05 (Tukey–Kramer’s test). Skm-PCs, skeletal muscle progenitor cells; SPARC, secreted protein acidic and rich in cysteine.

To determine the endocytic pathway of SPARC internalization, the expression of C-terminal binding protein-1/brefeldinA-ADP-ribosylated substrate (BARS), a critical mediator of macropinocytosis (Liberali *et al*., 2008; Haga *et al*., 2009; Gu *et al*., 2011), clathrin heavy chain (Cltc), and caveolin1 (Cav1) were suppressed by siRNA before addition of Alexa-SPARC into the media used for Skm-PCs from young rats (Fig. 3). Knockdown efficiency was confirmed by qPCR (Fig. 3A), which demonstrated more than 90% suppression. Under these conditions, the amounts of internalized SPARC in the siBARS and siCav1 groups were comparable with those for the control, whereas the amount of SPARC internalized by Skm-PCs treated with siCltc was suppressed by approximately 90% (Fig. 3B and C). Therefore, we suggest that itga5-dependent SPARC internalization is regulated by clathrin-mediated endocytosis. We evaluated the cell number by counting Hoechst-positive nuclei in each group; control, 29 ± 1.8; siBARS, 29.6 ± 2.9; siCav1, 27.6 ± 4; siCltc, 25.2 ± 0.9 cells per field [means ± SEM (*n* = 5)]. There was no significant difference in the number of cells among each treatment (Tukey–Kramer’s test). Thus, it does not seem that siRNA treatment caused cell death or inhibited cell proliferation.

### Internalized SPARC localizes to Rab7-positive late endosomes

**Figure 4:**
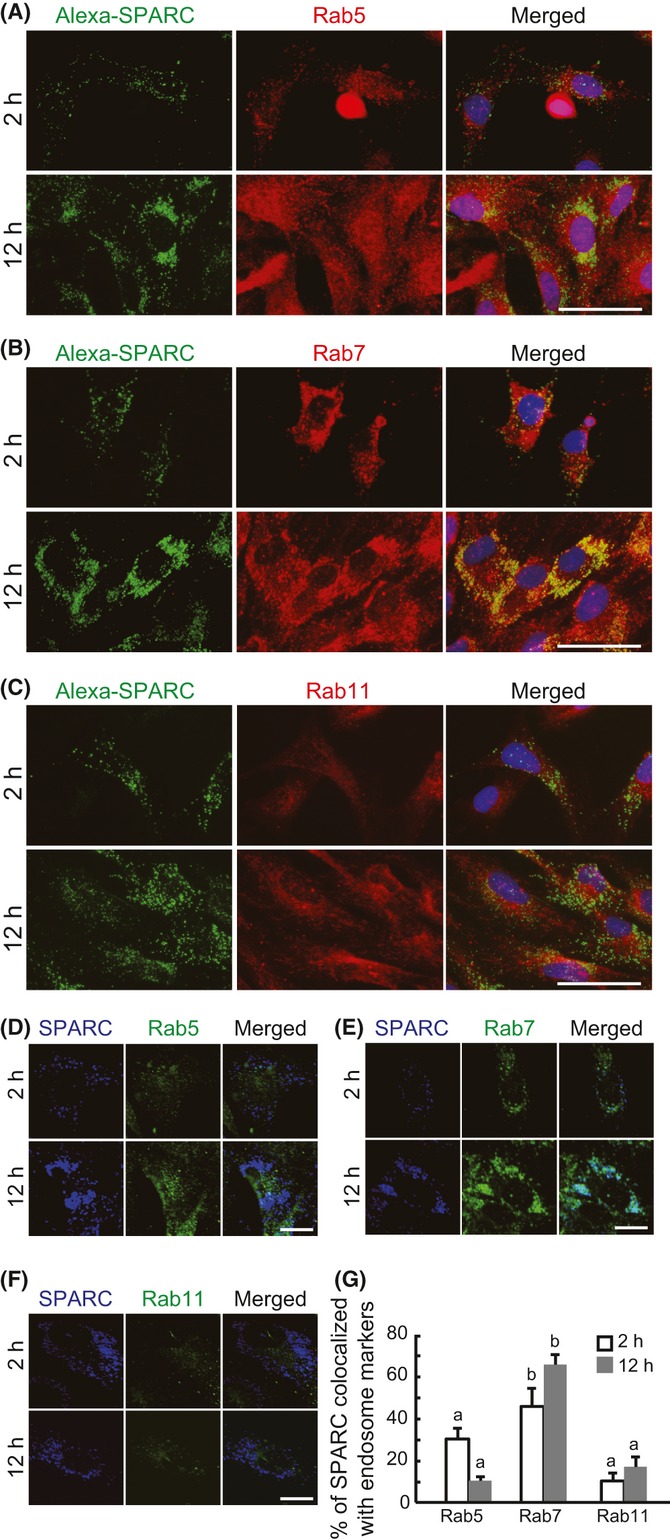
Identification of the endosome of internalized SPARC. Skm-PCs from young rats were incubated with Alexa-SPARC (green) for 2 or 12 h and immunostained with antibodies for the endosome markers, Rab5 (A, red), Rab7 (B, red), and Rab11 (C, red). Nuclei were visualized with Hoechst 33258 dye (blue). Scale bar = 50 μm. Confocal images of Skm-PCs derived from young rats incubated with Alexa-SPARC (blue) for 2 or 12 h and stained with anti-Rab5 (D, green), anti-Rab7 (E, green), and Rab11 (F, green). Scale bar = 20 μm. (G) SPARC co-localization with endosome markers in Skm-PCs from young rats were graphed by ImageJ. Error bars represent means ± SEM (*n* = 10 cells). Bars sharing the same letters are not significantly different. *P* < 0.05 (Tukey–Kramer’s test). Skm-PCs, skeletal muscle progenitor cells; SPARC, secreted protein acidic and rich in cysteine.

To identify internalized SPARC endosomes, we performed immunostaining with antibodies to early endosome marker Rab5, late endosome marker Rab7, and recycling endosome marker Rab11 (Fig. 4). As shown in Fig. 4A–C, internalized SPARC was strongly co-stained with anti-Rab7 antibody 12 h after addition of Alexa-SPARC. Confocal analysis revealed that at 2 h after addition of Alexa-SPARC, SPARC was co-localized with Rab5 and Rab7, but not Rab11 (Fig. 4D–F). At 12 h, most of the Alexa-SPARC was co-localized with Rab7, rather than Rab5 and Rab11. Quantification of co-localization of internalized SPARC and endosome markers confirmed these results (Fig. 4G). These data indicate that internalized SPARC is internalized via early endosomes and most is transported to late endosomes, not through the integrin recycling route.

### Cltc and Rab7 vary with age in MyoD-negative Skm-PCs

**Figure 5:**
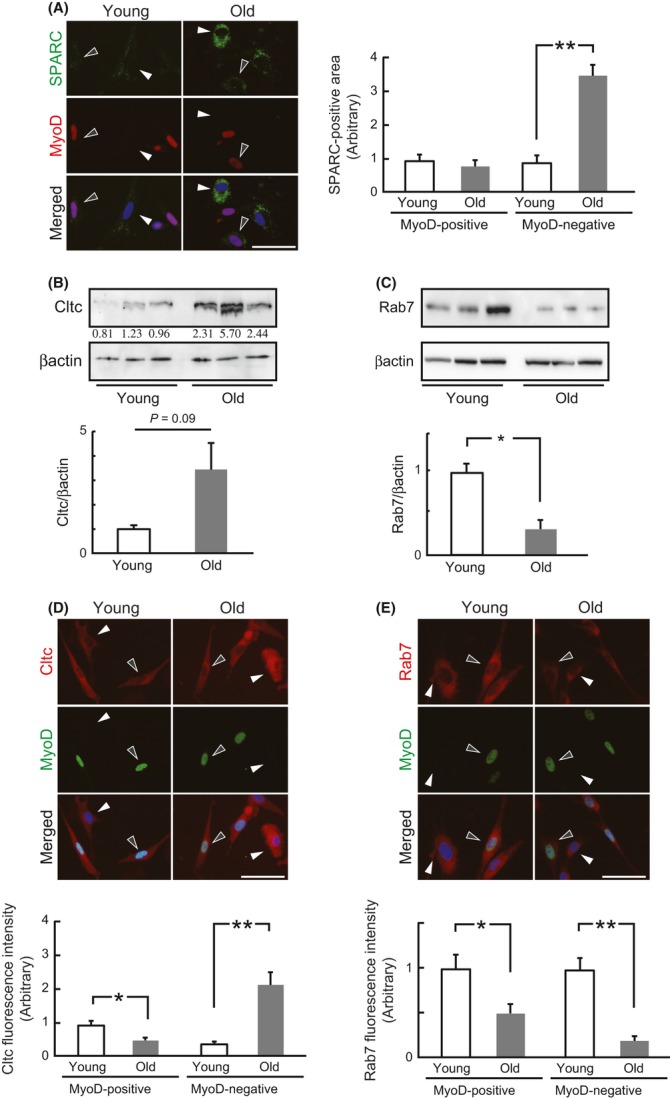
The SPARC internalization pathway in Skm-PCs from young and old rats. (A) Skm-PCs derived from young and old rats were incubated with Alexa-SPARC (green) and immunostained with anti-MyoD (red). Representative photographs of MyoD-positive and MyoD-negative cells in Skm-PCs from young and old rats are shown in the left panel. Black arrowheads indicate MyoD-positive and white arrowheads indicate MyoD-negative cells, respectively. Scale bar = 50 μm. SPARC fluorescence per cell was quantified by ImageJ and graphed (right). Error bars represent means ± SEM (*n* = 10 cells). **, *P* < 0.01. The expression of Cltc (B) and Rab7 (C) was compared in Skm-PCs from young and old rats at 2 days after isolation. Graphed data are expressed as means ± SEM (*n* = 3). *, *P* < 0.05. Co-immunostaining of MyoD and Cltc (D) or Rab7 (E) and quantification of Skm-PCs from young and old rats at 2 days after isolation. Representative photographs of MyoD and Cltc or Rab7 in Skm-PCs from young and old rats are shown above. Black arrowheads indicate MyoD-positive and white arrowheads indicate MyoD-negative cells, respectively. Scale bar = 50 μm. Fluorescence intensity was quantified by ImageJ and graphed (below). Error bars represent means ± SEM (*n* = 10 cells). *, *P* < 0.05, **, *P* < 0.01. Skm-PCs, skeletal muscle progenitor cells; SPARC, secreted protein acidic and rich in cysteine.

We then investigated whether these SPARC internalization factors change with age. Immunostaining of MyoD, a marker for just-activated satellite cells and their proliferating progeny, which are subpopulations of myogenic cells, revealed that the amount of internalized SPARC did not differ between myogenic Skm-PCs from young and old rats, whereas MyoD-negative Skm-PCs from old rats internalized about four times the amount of Alexa-SPARC internalized by those from young rats (Fig. 5A).

Immunoblotting revealed age-related changes in Cltc and Rab7 expression; although the difference was not significant (*P* = 0.09), all Skm-PCs from old rats showed a more than twofold increase in Cltc expression in comparison with that in young rats (Fig. 5B). Rab7 was significantly reduced in Skm-PCs from old rats (Fig. 5C).

Co-immunostaining of MyoD and Cltc revealed decreased expression of Cltc in MyoD-positive cells from old rats, whereas expression of Cltc was about fivefold greater in MyoD-negative cells from old rats (Fig. 5D). Expression of Rab7 was lower in MyoD-positive and MyoD-negative cells, and the age-related decline in MyoD-negative cells was more obvious than that in MyoD-positive cells (Fig. 5E). These results suggest that the age-related alteration in the SPARC pathway is likely to occur in MyoD-negative Skm-PCs.

### Clathrin attenuates and Rab7 enhances the anti-adipogenic effect of SPARC

**Figure 6:**
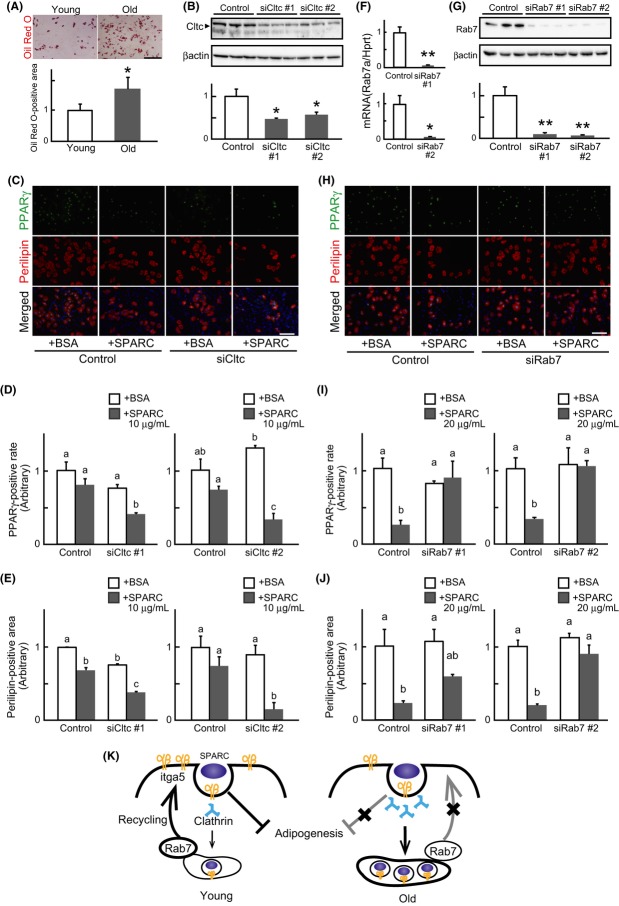
Clathrin and Rab7 are key regulators of the anti-adipogenic effect of SPARC. (A) Representative images of Oil Red-O staining in Skm-PCs derived from young and old rats were shown above, and the data are graphed below as means ± SEM (*n* = 3). *, *P* < 0.05. (B) siCltc silencing efficiency was evaluated by immunoblotting in Skm-PCs from young rats 2 days after siRNA transfection. Data are expressed as the means ± SEM (*n* = 3). *, *P* < 0.05 (Dunnett’s test). (C) Immunostaining of PPARγ (green) and perilipin (red) was performed, and representative photographs of Skm-PCs from young rats in adipogenic differentiation media for 4 days with 10 μg/mL BSA or SPARC after control siRNA (control, above) or siCltc (below) transfection are shown. Nuclei were visualized with Hoechst 33258 dye (blue). Scale bar = 50 μm. The rate of PPARγ-positive cells vs. total cell number (D) and positive area of perilipin divided by cell number quantified by ImageJ (E) were graphed and compared. Data are expressed as means ± SEM (*n* = 3). Bars sharing the same letters are not significantly different. *P* < 0.05 (Tukey-Kramer’s test). (F) The efficiency of Rab7-targeted siRNA (siRab7) was evaluated by qPCR in Skm-PCs from young rats 2 days after siRNA transfection. Error bars represent means ± SEM (*n* = 3). *, *P* < 0.05, **, *P* < 0.01. (G) siRab7 silencing efficiency was evaluated by immunoblotting in Skm-PCs from young rats 2 days after siRNA transfection. Data are expressed as the means ± SEM (*n* = 3). **, *P* < 0.01 (Dunnett’s test). (H) Immunostaining of PPARγ (green) and perilipin (red) was performed, and representative photographs of Skm-PCs from young rats in adipogenic differentiation media for 4 days with 20 μg/mL of BSA or SPARC after control siRNA (control, above) or siRab7 (below) transfection are shown. Nuclei were visualized with Hoechst 33258 dye (blue). Scale bar = 50 μm. The rate of PPARγ-positive cells vs. total cell number (I) and positive area of perilipin divided by cell number quantified by ImageJ (J) were graphed. Data are expressed as means ± SEM (*n* = 3). Bars sharing the same letters are not significantly different. *P* < 0.05 (Tukey-Kramer’s test). (K) Proposed model of age-related SPARC resistance. In the young rats, SPARC binds itga5 and inhibits adipogenesis and then is internalized with clathrin. Rab7 recycles itga5 to the cell surface, and extracellular SPARC can bind itga5 again. In the old rats, itga5 decreases, and clathrin-mediated endocytosis is enhanced so that the anti-adipogenic effect of SPARC through itga5 on the cell surface is reduced. Loss of Rab7 delays itga5 recycling, which can reduce responsiveness to SPARC with age. Skm-PCs, skeletal muscle progenitor cells; SPARC, secreted protein acidic and rich in cysteine.

Similar to the results obtained in mice (Taylor-Jones *et al*., 2002), we confirmed that the adipogenic potential of Skm-PCs from old rats increased compared with those from young (Fig. 6A). We previously reported that Skm-PCs from old rats become refractory to the anti-adipogenic effect of SPARC. Additionally, from the above findings, Cltc is shown to be involved in SPARC internalization, and increased Cltc expression was observed only in MyoD-negative Skm-PCs from old rats. Typically, in our culture of Skm-PCs, 75–85% are MyoD-positive cells, and 15–25% are capable of differentiating into adipocytes upon adipogenic stimulation (data not shown). Thus, most of the MyoD-negative Skm-PCs could be regarded as adipogenic cells. From these, we hypothesized that Cltc-mediated SPARC internalization has a negative impact on the anti-adipogenic effect of SPARC, and if so, it would be expected that suppression of Cltc in Skm-PCs may result in enhancing the anti-adipogenic effect of SPARC. To examine this, we performed loss-of-function analysis by using Cltc-targeted siRNA in Skm-PCs from young rats. Approximately 50% of Cltc protein expression was suppressed by siRNAs (Fig. 6B), and immunostaining of PPARγ and perilipin, markers of adipocytes, showed that the anti-adipogenic effect of SPARC was enhanced by Cltc suppressions (Fig. 6C–E). These results indicate that Cltc-mediated SPARC internalization negatively regulates the anti-adipogenic effect of SPARC and suggest that increased expression of Cltc in Skm-PCs from old rats is strongly associated with their refractoriness to the effect of SPARC.

Next, we examined the role of Rab7 in anti-adipogenic effect of SPARC using Rab7-targeted siRNA. Approximately 95% of Rab7 gene (Fig. 6F) and 90% of protein (Fig. 6G) expression were suppressed by siRNAs, and Rab7 suppressions weakened the effect of SPARC (Fig. 6H–J). These results indicate that Rab7 plays a role in the expression of anti-adipogenic effect of SPARC and suggest that decreased Rab7 expression in Skm-PCs from old rats is also related to their refractoriness to the effect of SPARC.

## Discussion

We previously reported that SPARC has both promyogenic and anti-adipogenic effects and that these effects on Skm-PCs decline with age (Nakamura *et al*., 2012). In the present study, age-related change in itga5-mediated SPARC internalization was not observed in MyoD-positive cells, suggesting that alterations in SPARC internalization pathway are unlikely to cause the age-related decrease in the promyogenic effect of SPARC. On the other hand, itga5-mediated SPARC internalization was enhanced in MyoD-negative cells from old rats, and this was accompanied by an increased expression of clathrin, which mediates SPARC internalization and negatively regulates the anti-adipogenic effect of SPARC. Therefore, the increased SPARC internalization may contribute to the age-associated decline in the responsiveness to the anti-adipogenic effect of SPARC in Skm-PCs.

This study clearly demonstrates that SPARC is internalized by Skm-PCs through an itga5-dependent pathway. In macrophages, SPARC is internalized through interaction with stabilin-1, a scavenger receptor that may act to clear extracellular SPARC (Kzhyshkowska *et al*., 2006). SPARC internalized by macrophages was first located in the early endosomes and was then found to move to Rab7-positive late endosomes, consistent with our findings. This study is the first to demonstrate that nonphagocytic cells other than macrophages similarly internalize SPARC through itga5, which is necessary for SPARC regulation of adipogenic differentiation.

Clathrin regulates various ligand–receptor-dependent endocytosis pathways such as those involving EGF and Wnt (Disanza *et al*., 2009), but its role differs between ligands. siRNA-mediated suppression of clathrin impairs Wnt3A signaling, suggesting that clathrin plays an essential role in Wnt internalization as a ‘signaling endosome’ (Blitzer & Nusse, 2006). In contrast, blocking EGF internalization by deletion of dynamin, a mediator of endocytic fission of coated pits, or knockdown of clathrin enhances EGFR autophosphorylation and sustains Akt stimulation, a downstream molecule in EGF signaling (Sousa *et al*., 2012), indicating that EGF signaling is terminated by endocytosis. In this study, the anti-adipogenic effect of SPARC was enhanced by clathrin suppression, suggesting that SPARC signaling is terminated by endocytosis. Clathrin is also a regulator of integrin; suppressing clathrin increases the amount of itga5 on the cell surface (Moravec *et al*., 2012). It is possible that age-related increases in the clathrin heavy chain enhance the termination of the anti-adipogenic effect of SPARC and decrease the amount of itga5 on the cell surface, which may contribute to the age-related decline in Skm-PC responsiveness to SPARC.

This study shows that Rab7 interacts with SPARC at the late internalization pathway and plays an essential role in SPARC responsiveness. Studies using a dominant-negative Rab7 revealed its role as a regulator of transport from early to late endosomes (Feng *et al*., 1995; Press *et al*., 1998); in addition, Rab7 is critical for the fusion of late endosomes to lysosomes (Bucci *et al*., 2000). A recent study demonstrated a possible new role of Rab7 in the recycling of membrane type 1-matrix metalloproteinase (MT1-MMP), as the delivery of internalized MT1-MMP back to the plasma membrane is dependent on Rab7. Interestingly, this study showed that MT-MMP1 and itga5 are co-localized and co-transported at the early stage of MT-MMP1 endocytosis. These observations and our results suggest that Rab7 plays a positive role in the recycling of molecules on the cell membrane from late endosomes; itga5 is also recycled from late endosomes by Rab7. If this is indeed the case, then age-related decline in Rab7 causes dysregulation in the cascade of itga5 recycling, which reduces the amount of itga5 on the cell surface.

Together with the current findings and our previous study (Nakamura *et al*., 2012), a proposed model for age-related resistance to SPARC in the adipogenesis of Skm-PCs is shown in Fig. 6K. In young rats, SPARC inhibits adipogenesis through itga5 on the cell surface, and its effect is terminated by clathrin-mediated endocytosis. In the late endosome, Rab7 recycles itga5 to the cell surface, maintaining responsiveness to SPARC. In old rats, the age-related decline in itga5 is accompanied by enhanced clathrin-mediated endocytosis and suppression of Rab7, which reduces the anti-adipogenic effect of SPARC on the surface and delays itga5 recycling, causing age-related changes in the responsiveness to the anti-adipogenic effect of SPARC. Age-related changes in SPARC internalization may be a target for preventing the intramuscular fat accumulation associated with aging in skeletal muscle.

## Experimental procedures

### Animals

Young (age 2–4 months) and old (age >21 months) male Wistar-Imamichi rats were used in this study. All animals were bred in our laboratory under controlled environmental conditions [23 °C with a 12 h: 12 h photoperiod (lights on at 0700)]. Animals were allowed *ad libitum* access to commercial chow (Lab MR-Breeder Standard Nihon Nosan Kogyo, Yokohama, Japan). All animal experiments were performed according to the Guide for the Care and Use of Laboratory Animals of the University of Tokyo and were approved by the Institutional Animal Care and Use Committee of the University of Tokyo.

### Skm-PC isolation and culture

Skeletal muscle-derived progenitor cells were isolated from the hindlimb muscles of rats according to published procedures (Yamanouchi *et al*., 2006, 2007). To examine adipogenic potential, Skm-PCs were cultured in Dulbecco’s modified Eagle’s medium (DMEM) containing 10% fetal bovine serum (FBS) and penicillin/streptomycin (10% FBS/DMEM) supplemented with insulin (1 μg/mL), dexamethasone (0.1 μg/mL), isobutylmethylxanthine (27.8 μg/mL), and troglitazone (10 μm, kindly donated by Daiichi Sankyo Co., Ltd., Tokyo, Japan) for 2 days, followed by culture for 2 days in 10% FBS/DMEM supplemented with insulin and troglitazone. Troglitazone is a PPARγ agonist and was used to maximize adipogenic potential of Skm-PCs. Recombinant human SPARC (120-36, PeproTech, Rocky Hill, NJ, USA) was added after transfection with SPARC-targeted siRNA. An equal amount of bovine serum albumin (BSA, Nacalai Tesque, Inc., Kyoto, Japan) was used as a control. Alexa labeling kits (Invitrogen, Carlsbad, CA, USA) were used for fluorescence labeling of BSA and recombinant SPARC according to the manufacturer’s protocol. After the Alexa fluorescence coupling reaction, proteins were resolved by SDS-PAGE and stained with Oriole™ fluorescent gel stain (Bio-Rad Corporation, Hercules, CA, USA). No degradation of the Alexa-conjugated SPARC was detected. Alexa-SPARC was added for 2, 12, or 24 h in 10% FBS/DMEM.

### RNA interference

RNA interference was carried out with siRNA duplexes designed to target rat SPARC as previously described (Nakamura *et al*., 2012). siRNAs targeted to integrin-α5 (itga5), C-terminal binding protein-1/BARS, clathrin heavy chain (Cltc), caveolin1 (Cav1), and Rab7 were purchased from Applied Biosystems/Ambion (Austin, TX, USA). Negative control siRNAs (Silencer Select Negative Control #1 or #2, Ambion) were also used. As commercially indicated, there are no differences between SPARC internalization and adipogenic potential of Skm-PCs in both negative controls (data not shown). Two or 3 days after plating, cells were transfected with siRNAs using Lipofectamine RNAi Max (Invitrogen) according to the manufacturer’s instructions. RNA samples were extracted from Skm-PCs 2 days after transfection, and gene silencing efficiency was confirmed by qPCR. The sense strand sequences of siRNAs were as follows: itga5; #1 (5′-cauucaauuugacagcaaatt-3′), #2 (5′-acauguacccaacucuauatt-3′), BARS; #1 (5′-gcacaguggagaugccuautt-3′) #2 (5′ -cagcggguuugacaacauatt-3′), Cltc; #1 (5′-caacaaguccgugaacgaatt-3′), #2 (5′-cauugaaguugguacaccatt-3′), Cav1; #1 (5′-cgacgacguggucaagauutt-3′), #2 (5′-cgcuugcugucuaccaucutt-3′), Rab7; #1 (5′-agaaguucaguaaccaguatt-3′), #2 (5′-cagacuuucugaccaaggatt-3′).

### Quantitative PCR (qPCR)

RNA was isolated using TRIzol (Invitrogen), and cDNA was synthesized using SuperScriptII reverse transcriptase (Invitrogen). Quantitative PCR was performed using Thunderbird qPCR (TOYOBO, Osaka, Japan) on a Light Cycler system (Roche, Basel, Switzerland). PCR was performed using the following primers: itga5 (forward, 5′-gcaccattcaatttgacagc-3′; reverse, 5′-ttgtactccacaggttcctcac-3′), hypoxanthine guanine phosphoribosyltransferase (Hprt) (forward, 5′-gaccggttctgtcatgtcg-3′; reverse, 5′-acctggttcatcatcactaatcac-3′), BARS (forward, 5′-gctcgcacttgctcaaca-3′; reverse, 5′-ctccaaccaaagctctcagg-3′), Cltc (forward, 5′-cgaatcctgaacaacctct-3′; reverse, 5′-catctattgatgttcgcagagc-3′), Cav1 (forward, 5′-aacgacgacgtggtcaaga-3′; reverse, 5′-cacagtgaaggtggtgaagc-3′), and Rab7 (forward, 5′-ggaggccatcaatgtgga-3′; reverse, 5′-cagctccacttccgtttcc-3′).

### Immunocytochemistry

For immunostaining, cultured Skm-PCs were fixed in 4% paraformaldehyde in phosphate-buffered saline (PBS) at room temperature (RT) for 15 min. After fixation, the cells were washed three times with PBS, followed by blocking with 5% normal goat serum/PBS containing 0.1% Triton-× 100 (Sigma) for 20 min at RT. Donkey serum was used in place of normal goat serum for detection of SPARC. Cells were incubated with primary antibodies (described below) overnight at 4 °C. After three washes with PBS, the cells were incubated with Alexa-Fluor-conjugated secondary antibodies (Invitrogen, 1:500 dilution) for 1 h at RT. Nuclei were counterstained with Hoechst 33258. Observations were made with a fluorescence microscope (BX50, Olympus, Tokyo, Japan) equipped with a digital camera (DP70, Olympus). To detect co-localization, confocal images were obtained using a Zeiss LSM510 system (Carl Zeiss Jena GmbH, Jena, Germany). For quantitative analysis of internalized SPARC of Skm-PCs (Fig. 1E) and itga5 expression (Fig. 2D), five different fields randomly chosen under the microscope using a 40 × objective were photographed and their fluorescence intensities were calculated as positive area by ImageJ software (ver.1.43, NIH, Bethesda, MD, USA), and then, the values were divided by the number of Hoechst-positive nuclei. For quantification of internalized SPARC (Figs 1D, 2E, 3C, and 5A) and expression of Cltc (Fig. 5D) and Rab7 (Fig. 5E), ten cells were randomly chosen from each group and the positive area of Alexa-SPARC, Cltc, or Rab7 was calculated in each cell by ImageJ software. For quantitative analysis of internalized SPARC and its co-localization with endosome markers (Fig. 4G), ten cells were randomly chosen from each group and the portion of SPARC-positive area that overlapped with endosome markers was calculated in each cell by ImageJ software. For quantification of PPARγ expression (Fig. 6D and I), the number of PPARγ-positive cells and Hoechst-positive nuclei in five different fields randomly chosen under the microscope using a 20 × objective was counted, and the portion of PPARγ-positive cells was calculated. For the quantification of perilipin expression (Fig. 6E and J), perilipin-positive areas in five different fields randomly chosen under the microscope using a 20 × objective were calculated by ImageJ software and the values were divided by the number of Hoechst-positive nuclei.

Primary antibodies and their species of origin were as follows: anti-SPARC (goat; R&D Systems, Minneapolis, MN, USA; 1:100 dilution), anti-itga5 (rabbit; AB1928, Millipore, Bedford, MA, USA; 1:1000 dilution), anti-Rab5 (rabbit; C8B1, Cell Signaling Technology, Beverly, MA; 1:100 dilution), anti-Rab7 (rabbit; clone D95F2, Cell Signaling Technology; 1:100 dilution), anti-Rab11 (rabbit; clone D4F5, Cell Signaling Technology; 1:50 dilution), anti-MyoD (mouse; clone A5.8, Novocastra, Newcastle upon Tyne, UK; 1:100 dilution), anticlathrin heavy chain (rabbit; clone D3C6, Cell Signaling Technology; 1:100 dilution), anti-PPARγ (rabbit; Santa Cruz Biotechnology, Santa Cruz, CA, USA; 1:200 dilution), and antiperilipin (rabbit; clone D1D8, Cell Signaling Technology; 1:500 dilution).

### Oil Red-O staining

Skm-PCs cultured in ADM for 4 days were fixed and stained with Oil Red-O mixture (2:3 mixture of 0.5% (w/v) Oil Red-O (Sigma) in 2-propanol and distilled water) for 10 min. For quantification of adipogenesis, five different fields randomly chosen under the microscope using a 10 × objective were photographed, and the areas stained with Oil Red-O were quantified using ImageJ software.

### Immunoblotting

Skm-PCs were lysed in sample buffer [0.5 m Tris–HCl, 10% glycerol, 1% sodium dodecyl sulfate (SDS), and 10% 2-mercaptoethanol]. Protein extracts were separated on SDS–polyacrylamide gels and transferred to polyvinylidene fluoride membranes. Protein was detected using anticlathrin heavy chain (1:1000) and anti-Rab7 (1:1000), followed by incubation with horseradish-peroxidase-labeled secondary antibody and ECL detection (GE Healthcare Biosciences, Piscataway, NJ, USA). Blots with anti-β-actin antibody (mouse monoclonal antibody, A1978, Sigma) were used as a loading control.

### Statistical analyses

Statistical differences between groups were evaluated using anova followed by Tukey–Kramer’s or Dunnett’s post hoc comparisons. Student’s *t*-test was used to examine differences between pairs of groups. *P* values < 0.05 were considered statistically significant.
